# Victimisation, Depression and Suicidal Ideation among Lesbian, Gay and Bisexual Adults in Malaysia

**DOI:** 10.21315/mjms2022.29.4.11

**Published:** 2022-08-29

**Authors:** Norhayati IBRAHIM, Magtum AEN, Noh AMIT, Zaini SAID, Ching Sin SIAU

**Affiliations:** 1 Center for Healthy Ageing and Wellness, Faculty of Health Sciences, Universiti Kebangsaan Malaysia, Kuala Lumpur, Malaysia; 2 Institute of Islam Hadhari, Universiti Kebangsaan Malaysia, Selangor, Malaysia; 3 Clinical Psychology Programme, Faculty of Health Sciences, Universiti Kebangsaan Malaysia, Kuala Lumpur, Malaysia; 4 Center for Community Health Studies, Faculty of Health Sciences, Universiti Kebangsaan Malaysia, Kuala Lumpur, Malaysia

**Keywords:** victimisation, depression, suicidal ideation, lesbian, gay, bisexual

## Abstract

**Background:**

Lesbian, gay and bisexual (LGB) individuals have been found to experience a high prevalence of psychological conditions. The present study aims to examine the relationship between victimisation, depression and suicidal ideation among LGB adults in Malaysia.

**Methods:**

This study used a cross-sectional design. We distributed the questionnaire online using snowball sampling and self-identified LGB adults responded to the survey. Scales on sexual minority-specific victimisation, depression and suicidal ideation were employed. The data were analysed using Spearman rank order correlation and Kruskal-Wallis tests.

**Results:**

The study included 220 adults in Malaysia (156 males and 64 females; 58 lesbian, 112 gays and 50 bisexuals). Ninety-two participants (41.8%) reported experiencing sexual minority-specific victimisation; 29 participants (13.2%), 35 participants (15.9%) and 23 participants (10.5%) were mildly, moderately and severely depressed, respectively and 44.0% participants were experiencing current suicidal ideation. The correlations were fair (*r_s_* = 0.30) between victimisation and depression, poor (*r_s_* = 0.16) between victimisation and suicidal ideation, and moderate (*r_s_* = 0.60) between depression and suicidal ideation. There were no differences between homosexual and bisexual participants in victimisation, depression and suicidal ideation.

**Conclusion:**

This study contributes to a better understanding of the relationship between victimisation, depression and suicidal ideation among LGB adults in Malaysia.

## Introduction

There is a consensus among researchers that victimisation represents a highly stressful experience for its victim (1–2). In examining the specific targets of victimisation, several studies have suggested that individuals perceived as sexual and gender minorities (i.e. lesbian, gay, bisexual and transgender [LGBT] individuals) are particularly vulnerable (3). Sexual and gender minority individuals had 3.74 higher odds of experiencing victimisation compared to heterosexual individuals (4) and were 2 times–9 times higher violent victimisation rates than heterosexuals (3). Sexual gender minority students were more likely to experience sexual victimisation compared to cisgender heterosexual male students (5). Sexual and gender minority adults also recorded more mental health issues such as depression and suicidal thoughts and behaviour. The prevalence of suicidal attempts among lesbian, gay and bisexual (LGB) adults in population studies was 11.0% and 20.0% in community surveys, compared to 4.0% among heterosexuals (6). Bisexual individuals consistently recorded higher prevalence of depression (7) and suicide attempts compared to heterosexuals (8). A study among Muslim lesbian women found that half of the participants were at least mildly depressed (9). In the Asian context, a recent study in ASEAN found that 23.5% of the LGBT university students reported severe depression and 40% reported the presence of suicidal ideation, and 2.8 higher odds of attempting suicide compared to heterosexual youth (10).

Victimisation is associated with compromised emotional health and suicidal ideation in its victims (11). Victimisation is linked to suicidal ideation and being drunk once in the past year among men who have sex with men in China (12). Males who experienced interpersonal violence were more likely to have been sexually abused as a child (13). Sexual/gender minority youth who experienced victimisation reported increased mental, physical and behavioural health issues (5).

Due to the close inter-relationship between sexual/gender minority status and victimisation, Meyer (14) proposed the Minority Stress Model. It posits that haemogenic structures that upheld heterosexism and homophobia created stressors (e.g. discrimination, stigma and victimisation), which could result in mental health issues and risky behaviour among those with minority status (14–15). Scandurra et al. (16) extended the Minority Stress Model to gender minorities as well and they found that Italian transgender individuals who experienced oppression, stigmatisation and discrimination reported increased mental health issues.

The health, social and psychological issues among sexual/gender minorities have been receiving increased attention in the West. An increasing number of studies in this area has also been conducted in Asia (17–19). However, the bulk of studies on this topic is still largely limited to the West or developed nations. An open and critical discussion about sexuality remains a taboo or is prohibited in most of the non-Western countries. As a result, studies related to sexual minorities from these countries are under-represented. In addition, the majority of studies on victimisation among the sexual/gender minority had focused on youth or adolescents (5). Since there is a very limited number of relevant studies in Malaysia, this study aimed to examine the prevalence of victimisation, depression and suicidal ideation, and the relationship between these variables among LGB adults in Malaysia.

## Methods

### Research Design

This study used a cross-sectional study design.

### Participants and Sampling Procedures

Malaysian adults aged 18 years old and above who self-identified as gay, lesbian or bisexual were included in this study. Those who refused to provide informed consent were excluded. A sample size of 115 was determined by assuming the proportion of expected LGB population prevalence of 4.5% (*P* = 0.045) (20), precision (*d* = 0.05) and 99% level of confidence. Assuming a non-response rate of 30%, the minimum number of participants that should be recruited was 150. Snowball sampling method was adopted by disseminating the questionnaire to LGB individuals whom the authors knew and to LGB-related groups. The participant information sheet stated the inclusion criteria (i.e. 18 years old and above, and self-identifying as LGB) so that only LGB adults would respond to the survey. Informed consent was obtained from participants before they proceeded to answer the online survey questions.

### Instruments

#### Sociodemographic Information

Information on age, gender, sexual identity, ethnicity, religion, highest education attained, monthly income and relationship status were obtained.

#### Sexual Minority-Specific Victimisation

Victimisation due to LGB status was measured by four items. These items assessed the frequency of participants being: i) teased or bullied; ii) hit or beaten up; iii) treated unfairly and iv) called bad names because of their sexual minority status during the past 12 months. Response was scored as 0 = never; 1 = once or twice; 2 = a few times or 3 = many times. A mean score of the four items was computed and used in the analyses. These four items have acceptable internal consistency reliability (α = 0.86) (21). In this study, the scale score yielded an acceptable internal consistency reliability of α = 0.72.

#### Beck Depression Inventory-2nd Edition

The Beck Depression Inventory-2nd edition (BDI-II) was administered to assess the frequency of participants’ depressive symptoms during past 2 weeks, including the day of participating in the survey. The 21 self-report items in the BDI-II measure the severity of depression in adults and adolescent aged 13 years old and older, with the assessment of symptoms corresponding to criteria for diagnosing depressive disorders listed in the American Psychiatric Association’s Diagnostic and Statistical Manual of Mental Disorders-4th edition (DSM-IV) (22): i) sadness; ii) pessimism; iii) sense of failure; iv) self-dissatisfaction; v) guilt, vi) punishment; vii) self-dislike; viii) self-accusations; ix) suicidal ideas; x) crying; xi) irritability; xii) social withdrawal; xiii) indecisiveness; xiv) body image change; xv) work difficulty; xvi) insomnia; xvii) fatigability; xviii) loss of appetite; xix) weight loss; xx) somatic preoccupation and xxi) loss of libido (23). The BDI-II has been used in various Western (24–25) and non-Western (26–27) studies to measure depression among LGB individuals. Its internal consistency reliability in these studies ranged from 0.82 to 0.92 (24–27). In this study, the scale score yielded an excellent internal consistency reliability of α = 0.92.

#### Beck Scale for Suicide Ideation

The Beck Scale for Suicide Ideation (BSS) was administered to participants in order to assess their suicide ideation for the past weeks, including the day of participating in the survey. The first five BSS items serve as a screen for suicide ideation. If a participant circled the zero statements in both Item 4 (indicating no active suicidal intention) and Item 5 (indicating avoidance of death if presented with a life-threatening situation), he or she was instructed to skip the next 14 BSS items, which address specific information about the participant’s plans and attitudes. Otherwise, the participant rated the next 14 BSS items. Every participant was asked to rate Item 20 and any participant who had previously attempted suicide was requested to rate Item 21 (28). The BSS has also been used in a number of Western (29) and non-Western (30) studies to measure suicidal ideation among LGB individuals. Its internal consistency reliability in these studies ranged from α = 0.81 to 0.95 (29–30). In this study, the scale score yielded an excellent internal consistency reliability of α = 0.97.

### Data Analysis

The data was analysed using IBM SPSS Statistics for Windows, version 26.0 (Armonk, NY: IBM Corporation). Descriptive analysis was used to describe the mean, standard deviation and percentage of the demographic data. The relationship between victimisation, depression and suicidal ideation among LGB adults in Malaysia was measured using the Spearman’s rank correlation test. Correlation coefficient of 0 was interpreted as no correlation; less than 0.3 as poor; 0.3–0.5 as fair; 0.6–0.8 as moderate and at least 0.8 as very strong (31). The differences between LGB adults on victimisation, depression and suicidal ideation were measured using the Kruskal-Wallis test.

## Results

### Sociodemographic Profile

There were 220 participants in this study. They were adults who age ranged from 18 years old to 54 years old, with mean age of 25.73 years old and standard deviation (SD) of 6.08. This sample comprised 156 (70.9%) males and 64 (29.1%) females. Most of the participants were Buddhists/Taoists (44.0%), had a Bachelor’s degree (42.7%) and a monthly income of RM999 or less (30.0%). Out of 220 participants, 112 (50.9%) self-identified as gay, 58 (26.4%) self-identified as lesbian and 50 (22.7%) self-identified as bisexual. With respect to relationship status, most of the participants were single (59.1%) (Table 1).

### Victimisation because of Social Orientation

**Figure 1:**
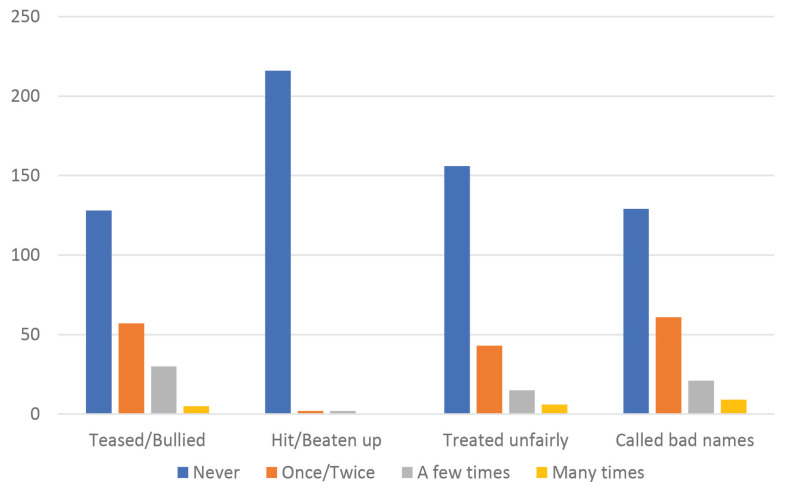
Frequency of victimisation because of sexual orientation in the past 12 months

Figure 1 illustrated that the type of victimisation which was most frequently experienced by the participants were being teased/bullied (41.8%), followed by being called bad names (41.4%) and treated unfairly (29.1%); the least experienced was being hit/beaten up (1.8%). In addition, it was observed that out of 220 participants, 128 (58.2%) of them had never experienced any form of victimisation and 92 (41.8%) of them had experienced at least one of the four forms of victimisation in the past 12 months.

### Level of Depression

Table 2 illustrated the levels of participants’ depression for the past 2 weeks. It was observed that out of 220 participants, 133 (60.5%) of them scored within minimal range for BDI-II, which formed the majority. This was followed by 35 (15.9%) participants who scored within the moderate range, 29 (13.2%) participants within the mild range and 23 (10.5%) participants within the severe range. The levels of depression had the mean score of 12.58 and SD of 10.78. The minimum score was 0 and the maximum score was 51 (Table 2).

### Results of Inferential Analyses

The result of normality test indicated that the variables in this study were not normally distributed. Skewness values of victimisation, depression and suicidal ideation were 1.65 (*z* = 10.05), 0.85 (*z* = 5.31) and 1.42 (*z* =8.66), respectively. Kurtosis values of the three variables were 3.07 (*z* = 9.39), 0.26 (*z* = 0.80) and 1.13 (*z* = 3.46), accordingly. All skewness and kurtosis values were within the normal distribution range except for the kurtosis value of depression. Because the assumptions of normality and random sample were not met, non-parametric procedures were used in analysing the data in this study, i.e. Spearman’s rank order correlation and Kruskal-Wallis test.

### Relationship between Victimisation, Depression and Suicidal Ideation

Table 3 showed the correlation between victimisation, depression and suicidal ideation. Spearman’s rank order correlation analyses showed the presence of a fair positive correlation between victimisation and depression (*r_s_* = 0.30; *P* < 0.01; *N* = 220). In addition, there was a poor positive correlation between victimisation and suicidal ideation (*r_s_* = 0.16; *P* < 0.05; *N* = 220). Finally, there was a moderate positive correlation between depression and suicidal ideation (*r_s_* = 0.60; *P* < 0.01; *N* = 220) (31).

### Differences in Median Scores between Lesbian, Gay and Bisexual Participants on Victimisation, Depression and Suicidal Ideation

A Kruskal-Wallis test indicated that there were no statistically significant differences in median scores for victimisation between lesbian (median = 2.0; interquartile range [IQR] = 3.0), gay (median = 1.0; IQR = 2.0) and bisexual (median = 1.0; IQR = 3.0) participants in this study.

In addition, the Kruskal-Wallis test suggested that there were no statistically significant differences in median scores for depression between lesbian (median = 12.0; IQR = 15.3), gay (median = 9.0; IQR = 16.0) and bisexual (median = 12.0; IQR = 18.3) participants in this study.

Furthermore, the Kruskal-Wallis test demonstrated that there were no statistically significant differences for suicidal ideation in median scores between lesbian (median = 0.0; IQR = 7.3), gay (median = 0.0; IQR = 8.0) and bisexual (median = 0.0; IQR = 8.0) participants in this study.

## Discussion

The main findings of this study were that 41.8% of the study participants reported experiencing sexual minority-specific victimisation in the past 12 months, 39.6% had at least mild depression and 44.0% experienced current suicidal ideation. In addition, LGB-specific victimisation was positively associated with depression and suicidal ideation.

The substantial prevalence of victimisation reported in this study was also found in a meta-analysis where a 56.0% prevalence of verbal harassment was reported among the LGB population in the United States (32). Bullying/teasing and verbal harassment were the most frequently reported victimisation types in this study. Unfortunately, studies on bullying victimisation among adults are rare, precluding meaningful inter-study comparisons. However, it is worth noting that between 20.0% and 25.0% of participants reported non-cyberbullying victimisation in a literature review on bullying victimisation in university/college samples (33). In another sample of working adults in Malaysia, 39.1% reported workplace bullying (34). These results indicate that bullying prevalence among sexual minorities may be higher than in university or work settings. Similarly, other forms of victimisation such as verbal/physical harassment and being treated unfairly due to sexual minority status was also prevalent among LGB students and youth (35–36). This study indicates that victimisation among the study participants continues to be a significant issue post-secondary education.

The high levels of current suicidal ideation and depression among the study participants is of concern. However, the results are comparable to that of Peltzer and Pengpid (10), where up to 40.0% of the undergraduates surveyed in ASEAN countries reported suicidal ideation and a nationally representative study on UK youth which found 45.3% of youth had suicidal ideation. The prevalence of depression among this sample is higher than that of 23.5% recorded by Peltzer and Pengpid (10). The suicidality and depression which the participants experienced may be explained by the victimisation they experienced, as LGB-specific victimisation was found to be moderately positively associated with depression and, to a lesser extent, suicidal ideation. Victimisation in general was associated with higher odds of depression (37) and suicidality (38). Among sexual minorities, Ferlatte et al.’s (39) research findings suggested that suicide ideation was positively associated with anti-gay marginalisation and workplace discrimination. In addition, the results of this study revealed that depression was strongly associated with suicidal ideation. Depression is a known risk of suicidal ideation and behaviour in the general population (40–41) and among sexual minorities (42–43).

The results of this study indicated there were no significant differences between the sexual minority-related victimisation experienced by the LGB adults. In other words, LGB adults in this study did not differ regarding their experiences of being teased or bullied, hit or beaten up, treated unfairly and called bad names because of their sexual orientation. Besides, the findings of this study suggested that there were no significant differences between levels of depression and suicidal ideation experienced by the LGB adults. This was similar with Shearer et al. (44), who found that while bisexual and questioning females were significantly more likely to experience depression than heterosexual females, there were no differences between lesbians, bisexual or questioning females in depression. However, a meta-analysis by Ross et al. (7) found the pooled prevalence of depression was higher among bisexuals compared to other sexual minorities. Blosnich and Bossarte (45), in investigating the self-injurious behaviours among college students (aged 18 years old–24 years old), similarly found that bisexuals had a greater prevalence of self-injurious and suicidal behaviours than lesbians and gays. The lack of difference in victimisation, depression and suicidal ideation levels between LGB individuals in this study could be due to the overall disapproval of non-heterosexual orientations in Malaysian law and religious practices (18).

## Limitations

Several limitations of this study were identified. First, because of the sensitivity of the subject-matter in Malaysia, participants were recruited by using the snowball-sampling method (non-probability sampling), as this method was more effective in accessing the potential participants. Due to the same reason of maintaining participant confidentiality, we were unable to objectively verify that the participants were indeed LGB individuals and had depended on the authenticity of their self-report. In addition, the questionnaire in the present study was quite lengthy (comprising of 63 items) and this may evoke boredom among participants, which may then lead to random responding towards the items. Even though the instrument Sexual Minority-Specific Victimisation had acceptable internal consistency reliability estimates, the validity of the instrument was not determined by the original authors (21). We did not attempt multivariate analysis and had only conducted non-parametric bivariate analyses due to the non-normality of the data distribution. Due to the stigma associated with sexual minority identity, participants in this study may have answered the questions in a socially desirable manner. Apart from depression and suicidal ideation, variables such as interpersonal relationship and coping skills could also be important factors in predicting the psychological outcomes of LGB individuals who were victimised.

The findings of this study have implications to the Malaysian culture and society. The high prevalence of victimisation among LGB adults in Malaysia indicates that there is an urgent need to raise awareness in society regarding the right to physical and psychological safety of each individual regardless of sexual orientation and to reduce stigma against LGB individuals which may have been the root cause of increased victimisation against them. Mental health professionals (psychiatrists, psychologists and counsellors) and social workers could initiate mental health services that are sensitive towards LGB-specific issues and to take into account the possibility of victimisation during assessment and treatment. In addition, there should be awareness campaigns that highlight the high prevalence of victimisation against LGB individuals.

## Conclusion

The findings of this study showed that there were significant positive correlations between victimisation, depression and suicidal ideation among LGB adults in Malaysia. Levels of victimisation, depression and suicidality were high but were comparable to studies conducted in other parts of the world. With such understanding, psychologists, public health professionals, public policy makers and relevant non-profit organisations can work towards designing effective prevention and intervention programmes in order to decrease the prevalence of victimisation and improve the quality of life among them. These steps could assist in not further marginalising individuals who identify as LGB in the Malaysian society.

## Figures and Tables

**Table 1:** Demographic characteristics of the sample (*N* = 220)

Characteristic	Frequency (%)
Age	
Mean	25.73
SD	6.08
Sex	
Male	156 (70.9)
Female	64 (29.1)
Sexual orientation	
Gay	112 (50.9)
Lesbian	58 (26.4)
Bisexual	50 (22.7)
Relationship status	
Single	130 (59.1)
In a committed relationship	78 (35.5)
Would rather not say/Other	12 (5.4)
Religious belief	
Islam	57 (25.9)
Christianity/Catholicism	16 (7.3)
Buddhism/Taoism	97 (44.0)
Hinduism	25 (11.4)
Atheism/Other	25 (11.4)
Highest level of education	
Primary/Secondary/Vocational/Technical School	61 (27.7)
Diploma/Certificate	51 (23.2)
Bachelor’s degree	94 (42.7)
Master’s degree	13 (5.9)
Other	1 (0.5)
Monthly income	
RM999 or less	66 (30.0)
RM1,000 to 1,999	32 (14.4)
RM2,000 to 2,999	38 (17.3)
RM3,000 to 3,999	38 (17.3)
RM4,000 to 4,999	23 (10.5)
RM5,000 and above	23 (10.5)

**Table 2:** Levels of participants’ depression for the past two weeks (*N* = 220)

Severity	Frequency
Minimal	133 (60.5)
Mild	29 (13.2)
Moderate	35 (15.9)
Severe	23 (10.5)

**Table 3:** Spearman’s rank order correlation between victimisation, depression and suicidal ideation

	Victimisation	Depression	Suicidal ideation
Victimisation	1		
Depression	0.30**	1	
Suicidal ideation	0.16**	0.60**	1

Notes:

** *P* < 0.01;

* *P* < 0.05
